# Economic burden of *Mycoplasma pneumoniae* infection in Jinhua City, China: a multicenter cross-sectional study

**DOI:** 10.3389/fpubh.2026.1835645

**Published:** 2026-06-01

**Authors:** Zhi-Ping Du, Ya-Ling Feng, Xiao Ma, Zhen Ye, Xiao-Cui Li, Juan-Juan He, Guo-Yong Jiang, Li Zhou, Zhi-Ping Long, Zhi-Feng Pang, Guang-Ming Zhang

**Affiliations:** 1 Department of Infectious Disease Prevention and Control, Jinhua Center for Disease Control and Prevention, Jinhua, China; 2 Regional Public Health Laboratory of Zhejiang Province, Jinhua Center for Disease Control and Prevention, Jinhua, China; 3 Department of Infectious Disease Prevention and Control, Lanxi Center for Disease Control and Prevention, Jinhua, China; 4 Jinhua Maternal and Child Health Hospital, Jinhua, China; 5 Department of Infectious Disease Prevention and Control, Yiwu Center for Disease Control and Prevention, Jinhua, China; 6 Sanjiang Subdistrict Community Health Service Center, Jinhua, China; 7 Jinhua Municipal People’s Hospital, Jinhua, China

**Keywords:** economic burden, medical costs, *Mycoplasma pneumoniae*, outpatients and inpatients, willingness to pay

## Abstract

**Background:**

*Mycoplasma pneumoniae* (MP) infection is a major cause of respiratory tract infections, particularly among children. However, quantitative evidence regarding its economic burden in China remains limited.

**Methods:**

A multicenter cross-sectional study was performed in Jinhua, China. Between October 2024 and March 2025, patients with laboratory-confirmed MP infection were enrolled using a multistage stratified random sampling approach. Data regarding direct, indirect, and intangible costs were collected via structured questionnaires and hospital information systems. Univariate analyses and generalized linear models (GLM) with a gamma distribution and log link function were employed to explore factors associated with the economic burden of MP infection.

**Results:**

A total of 582 patients were included, comprising 372 outpatients and 210 inpatients. The mean total cost per inpatient (¥6,289.21) was 7.69 times higher than that of outpatients (¥817.66). Among outpatients, direct and indirect medical costs contributed almost equally to the total expenditure, whereas inpatient costs were dominated by direct medical expenses. The median intangible cost was ¥5,000 for outpatients and ¥8,000 for inpatients. GLM analysis showed that prolonged disease duration, higher household income, and payment method were significantly associated with increased economic burden (*p* < 0.05). Among inpatients, chronic comorbidities were also a significant determinant (*p* < 0.05). Total costs increased progressively with longer disease duration.

**Conclusion:**

*Mycoplasma pneumoniae* infection imposes a considerable economic burden on patients in China, particularly among children, with cost structures differing between outpatient and inpatient settings. Early diagnosis, improved outpatient reimbursement policies, and future MP vaccine development may help reduce the economic burden.

## 1 Introduction

*Mycoplasma pneumoniae* (MP) is a common respiratory pathogen and an important cause of lower respiratory tract infections worldwide (1, 2). The pathogen is primarily transmitted through respiratory droplets or close contact, leading to inflammation of the respiratory tract and lungs (2). In severe cases, MP infection may cause extrapulmonary complications involving multiple organ systems. Smokers, infants, young children, and immunocompromised individuals are considered particularly susceptible populations (3).

MP infections occur globally throughout the year and are characterized by periodic epidemic cycles. Epidemiological evidence suggests epidemic intervals of approximately 1–3 years in Europe and Israel and 3–5 years in Asia (4, 5). In 2023, France reported a large outbreak mainly affecting children under 15 years of age, while Vietnam reported a pediatric outbreak in which nearly half of the cases carried macrolide-resistance–associated mutations (6, 7). The first prospective surveillance study of MP infection conducted by the European Society for Clinical Microbiology and Infectious Diseases (ESCMID) Study Group for Mycoplasma and Chlamydia (ESGMAC MAPS) reported an extremely low incidence of 0.82% between April 2022 and March 2023. However, since late 2022, several countries—including Denmark, Wales, France, and Singapore—have reported clear signs of resurgence. Detection rates increased rapidly to 4.12% between April and September 2023 (8, 9), indicating that MP infection has once again become an important global public health concern.

In China, a substantial increase in respiratory infections among children has been observed since October 2023. Surveillance data indicate that the nucleic acid detection rate of MP among pediatric patients reached 40.43%, which is markedly higher than the 5.59% observed in adults (10). These findings highlight the considerable disease burden associated with MP infection in China, particularly among children. Previous retrospective studies have also demonstrated the sustained endemicity of MP infection. The positivity rate of MP immunoglobulin M (IgM) among pediatric outpatients and emergency patients ranged from 7.80 to 10.12%, whereas the rate among hospitalized children ranged from 27.18 to 30.10% (11). A study conducted at Beijing Chaoyang Hospital from 2006 to 2016 reported that MP accounted for 37.5% of pneumonia cases (12), while research from Chengdu between 2014 and 2020 found that MP represented 18.7% of pathogens detected in lower respiratory tract infections (13).

The MP infection poses a significant threat to child health, and its cyclical epidemic patterns combined with increasing antimicrobial resistance further exacerbate the overall disease burden (14–16). Although numerous studies have investigated the epidemiological characteristics of MP infection (17, 18), quantitative evidence regarding its economic burden remains limited. Existing studies have primarily focused on direct medical costs, whereas indirect medical costs related to productivity loss and caregiving, as well as intangible costs reflecting physical suffering and psychological distress, have rarely been systematically assessed.

Furthermore, few studies have compared the economic burden between outpatient and inpatient settings, despite their distinct patterns of healthcare utilization and cost composition. Evidence from China is particularly scarce, especially regarding the role of medical insurance coverage and payment mechanisms in shaping the household economic burden.

Therefore, this multicenter study aimed to comprehensively quantify the direct, indirect, and intangible economic burden of MP infection, compare cost structures between outpatient and inpatient populations, and examine the influence of medical insurance payment patterns within the Chinese healthcare system.

## 2 Sources and methods

### 2.1 Study design

This multicenter cross-sectional study was conducted to assess the economic burden of MP infection and its associated determinants among outpatient and inpatient populations.

### 2.2 Sample size calculation

The required sample size was calculated using the single-sample proportion formula: *n = (U_α/2_)^2^PQ/δ^2^*, where the estimated prevalence was set at *p* = 20%, *Q* = 1 − *p* = 80%, the significance level was 𝛼=0.05 (two-sided), corresponding to a standard normal deviate of *U_𝛼/2_* = 1.96 and the allowable margin of error was 𝛿=0.04. Based on these parameters, the minimum required sample size was calculated to be 384 participants. To improve the precision and representativeness of the estimates, a larger sample than the minimum required was ultimately included. The estimated prevalence of 20% was based on previously reported detection rates of MP among patients with respiratory tract infections in China, which ranged from 18 to 30% in several hospital-based studies. The minimum sample size was calculated to estimate overall population parameters. Subgroup analyses of outpatients and inpatients were conducted to explore potential heterogeneity in economic burden and associated factors; therefore, the study was not specifically powered for subgroup-specific multivariable analyses, and these subgroup analyses should be considered exploratory in nature.

### 2.3 Study sites

A multistage stratified random sampling approach was adopted to ensure sample representativeness. In the first stage, study sites were selected from Yiwu City, Lanxi City, and Wucheng District, including secondary-level hospitals and above. In the second stage, patients were stratified by age group (0–5, 6–14, 15–59, and ≥60 years) and type of medical care (outpatient or inpatient). The total sample size was allocated across the four age strata in a ratio of 3:7:7:2. This allocation was based on the expected age distribution of MP infections reported in previous epidemiological studies in China, in which school-aged children and working-age adults accounted for a large proportion of cases (11, 13). The survey was conducted between October 2024 and March 2025. To improve representativeness, the final sample size within each stratum was proportionally adjusted according to the actual distribution of eligible MP cases in participating hospitals while maintaining the predefined allocation ratio as far as feasible.

### 2.4 Study participants

The sampling frame consisted of all patients with laboratory-confirmed MP infection who met the inclusion criteria and were recorded in the hospital information systems (HIS) of participating institutions during the study period. Within each stratum, participants were selected using simple random sampling with computer-generated random numbers. Sampling was conducted by trained personnel from local Centers for Disease Control and Prevention, independent of clinical teams, to minimize selection bias. Standardized procedures and quality control measures were implemented to ensure consistency across study sites. If a selected individual declined participation, a replacement was randomly selected from the same stratum using the same randomization procedure to maintain sample size and representativeness. Inclusion criteria were: (1) laboratory confirmation of MP infection, defined as a single serum antibody titer ≥1:160, a fourfold or greater increase in antibody titer between paired serum samples during the course of illness, or a positive nucleic acid test for MP; (2) voluntary participation by the patient or a legal guardian with written informed consent and the ability to complete the questionnaire. A total of 582 cases were investigated on site. It should be noted that this study did not systematically screen for other respiratory pathogens; therefore, co-infections could not be excluded, which may lead to potential overestimation of the economic burden attributable solely to MP infection.

### 2.5 Survey methodology

Based on the existing Economic Burden Questionnaire for Patients with Pulmonary Diseases and tailored to the clinical and epidemiological characteristics of MP infection, the MP Infection Economic Burden Survey Questionnaire was independently developed for this study. The questionnaire has not been previously published or validated elsewhere. An English version is provided as a Supplementary file. All investigators received standardized training and were required to pass a competency assessment prior to participation. Data collection was conducted using a one-to-one interview approach, combining face-to-face interviews in hospital wards with telephone follow-ups when necessary.

### 2.6 Survey content and variable definition

Socio-demographic variables included sex, age, educational attainment, marital status, occupation, household income, and type of medical insurance coverage. Direct medical costs included consultation and comprehensive service fees for the current outpatient visit or hospitalization, diagnostic and laboratory examination costs, treatment-related expenses, pharmaceutical costs, and medical consumables. Data on direct medical costs were primarily obtained from HIS. Indirect medical costs comprised transportation expenses incurred by the patient and accompanying caregiver during medical visits or hospitalization; additional nutritional expenditures; accommodation and meal costs; expenses for self-purchased medications; productivity losses due to work absence attributable to the patient’s illness; and productivity losses incurred by caregivers due to patient care. Productivity loss was estimated using the human capital approach. The number of workdays lost due to illness or caregiving was multiplied by the average daily wage of the patient or caregiver. For individuals without formal employment, the average daily wage of the local population was used as a proxy. Occupation was categorized into student, worker/farmer, and other occupations, with the latter including public administration and institutions, retirees, freelance workers, and individuals with occupations not otherwise specified. Intangible costs referred to the physical suffering and psychological distress experienced by patients and their families as a result of MP infection. These costs were quantified using the willingness-to-pay (WTP) approach. Before asking the WTP question, investigators explained the concept using standardized examples to ensure that respondents understood the hypothetical scenario. For pediatric patients, the questionnaire was completed by parents or legal guardians who were responsible for medical decision-making. Respondents were then asked: “What is the maximum amount you would be willing to pay to completely avoid the physical suffering and psychological distress caused by this illness?” The primary outcome variables included direct medical costs, indirect medical costs, total costs, and intangible costs. Direct medical costs, indirect medical costs, and total costs were included in multivariable GLM analyses, whereas intangible costs were analyzed descriptively because of their subjective and non-market nature.

### 2.7 Bias control

To minimize information bias, all investigators underwent standardized training and used a uniform questionnaire. Recall bias related to indirect cost estimation was reduced by conducting interviews as close to the illness episode as possible and supplementing missing information through medical record review and follow-up telephone interviews. After rigorous data quality assessment, including logical consistency checks and handling of missing values, all 582 cases were included in the final analysis.

### 2.8 Statistical analysis

Data were double-entered and validated using EpiData3.1, and statistical analyses were performed using SPSS23.0. Categorical variables were summarized as frequencies and percentages and compared using the Chi-square test. Continuous variables with non-normal distributions were described as medians and interquartile ranges (IQRs) and compared using the Wilcoxon rank-sum test for two-group comparisons and the Kruskal–Wallis H test for multiple-group comparisons. Generalized linear models (GLM) with a gamma distribution and log link function were applied to identify factors associated with the economic burden of MP infection. Variables with *p* < 0.10 in univariate analyses, together with variables considered clinically relevant based on previous literature and expert knowledge, were included in the multivariable GLM analyses. Multicollinearity among independent variables was assessed using variance inflation factors (VIF), with VIF > 5 indicating potential collinearity. Model fit was evaluated using deviance statistics and Pearson chi-square goodness-of-fit tests. Results were presented as adjusted cost ratios [*Exp(β)*] with 95% confidence intervals (*CIs*). All statistical tests were two-sided, and a *p* < 0.05 was considered statistically significant.

## 3 Results

### 3.1 Characteristics of the study population

A total of 582 patients with laboratory-confirmed MP infection were included in the analysis, comprising 372 outpatients (63.92%) and 210 inpatients (36.08%). Significant differences between outpatient and inpatient groups were observed in annual *per capita* household income, payment method, and the presence of chronic diseases (*p* < 0.05). Inpatients were more likely to have higher household income levels, use combined medical insurance and out-of-pocket payment methods, and present with underlying chronic conditions. No statistically significant differences were found between outpatients and inpatients with respect to sex, age distribution, occupation, antibiotic use, or disease duration (*p* > 0.05, Table 1).

**Table 1:** General demographic characteristics of the study population [*n* (%)].

Variables	*n*	Outpatients	Inpatients	*χ*^2^	*P*
Gender				2.543	0.111
Male	260	157(60.38)	103(39.62)		
Female	322	215(66.77)	107(33.23)		
Age				3.268	0.352
0–5 years	90	61(67.78)	29(32.22)		
6–14 years	216	140(64.81)	76(35.19)		
15–59 years	216	129(59.72)	87(40.28)		
≥60 years	60	42(70.00)	18(30.00)		
Occupation				7.156	0.067
Worker and Farmers	85	44(51.76)	41(48.24)		
Students	223	143(64.13)	80(35.87)		
Scattered Children and Kindergarten Children	109	72(66.06)	37(33.94)		
Others	165	113(68.48)	52(31.52)		
*Per capita* household income				14.054	0.003
¥0–50,000	250	141(56.40)	109(43.60)		
¥50,001–100,000	234	170(72.65)	64(27.35)		
¥100,001-150,000	73	46(63.01)	27(36.99)		
≥¥150,001	25	15(60.00)	10(40.00)		
Payment Method				61.836	<0.001
Medical Insurance	295	230(77.97)	65(22.03)		
Out-of-pocket	19	16(84.21)	3(15.79)		
Medical insurance + out-of-pocket	268	126(47.01)	142(52.99)		
Antibiotics				0.012	0.912
Yes	428	273(63.79)	155(36.21)		
No	154	99(64.29)	55(35.71)		
Chronic disease				6.901	0.009
Yes	72	36(50.00)	36(50.00)		
No	510	336(65.88)	174(34.12)		
Duration of illness				4.229	0.376
0–5 days	126	87(69.05)	39(30.95)		
6–10 days	306	187(61.11)	119(38.89)		
11–15 days	76	48(63.16)	28(36.84)		
16–20 days	39	24(61.54)	15(38.46)		
≥21 days	35	26(74.29)	9(25.71)		

### 3.2 Economic burden of outpatients

The total economic burden among outpatients with MP infection was ¥304,167.71, with a mean total cost of ¥817.66 per patient. Direct and indirect medical costs accounted for 49.20 and 50.80% of total costs, respectively. Diagnostic and laboratory examinations constituted the largest component of direct medical costs (20.71%), while productivity loss due to work absenteeism dominated indirect medical costs (48.38%). The average medical insurance reimbursement was ¥303.19, covering 75.36% of direct medical costs, whereas out-of-pocket expenditure accounted for 11.71% of total costs (Table 2).

**Table 2:** Economic burden categories for outpatients (¥).

Cost category	Subcategory	Cost	Percentage (%)	*Per capita*
Direct medical costs	Comprehensive service fees	15183.10	4.99	40.81
	Diagnostic and laboratory services	62994.90	20.71	169.34
	Therapeutic	13766.00	4.53	37.01
	Pharmaceuticals	55198.5	18.15	148.38
	Consumables	2520.10	0.83	6.77
Indirect medical costs	Transportation expenses	4823.20	1.59	12.97
	Loss of earnings	147146.91	48.38	395.56
	Self-purchased medication expenses	2535.00	0.83	6.81
Total costs	Total	304167.71	100.00	817.66
	Medical insurance	112788.10	37.08	303.19
	Out-of-pocket	35624.60	11.71	95.77

### 3.3 Economic burden of inpatients

Inpatients incurred a total economic burden of ¥1,320,734.91, with a mean cost of ¥6,289.21 per patient—7.69 times higher than that of outpatients. Direct medical costs accounted for 79.07% of total costs, while indirect medical costs accounted for 20.93%. Diagnostic and laboratory fees constituted the largest component of direct medical costs (33.83%), followed by comprehensive service fees (20.53%) and medications (15.83%). Indirect medical costs were mainly attributable to productivity loss (13.68%). The average insurance reimbursement was ¥3,653.61, covering 73.47% of direct medical costs, while out-of-pocket expenditure averaged ¥1,202.68 per patient (19.12% of total costs, Table 3).

**Table 3:** Economic burden categories for inpatients (¥).

Cost category	Subcategory	Cost	Percentage (%)	*Per capita*
Direct medical costs	Comprehensive service fees	271210.40	20.53	1291.48
	Diagnostic and laboratory services	446829.50	33.83	2127.76
	Therapeutic	83305.70	6.31	396.69
	Pharmaceuticals	209013.20	15.83	995.30
	Consumables	33979.80	2.57	161.81
Indirect medical costs	Transportation expenses	3725.30	0.28	17.74
	Loss of earnings	180738.01	13.68	860.66
	Meal expenses	56787.00	4.30	270.41
	Nutrition expenses	20146.00	1.53	95.93
	Caregiver fees	2300.00	0.17	10.95
	Self-purchased medication expenses	12700.00	0.96	60.48
Total costs	Total	1320734.91	100.00	6289.21
	Medical insurance	767257.70	58.09	3653.61
	Out-of-pocket	252562.40	19.12	1202.68

### 3.4 Intangible economic burden

Based on the WTP method, the median intangible economic burden was ¥5,000 for outpatients and ¥8,000 for inpatients. The intangible burden among inpatients was significantly higher than that among outpatients (*p* < 0.05, Table 4). WTP values were not included in the multivariate GLM analyses due to their subjective nature and potential dependence on individual economic capacity, which may violate model assumptions and reduce interpretability.

**Table 4:** Intangible economic burden on patients (¥).

Patient category	*Per capita* intangible cost	Interquartile Range		*Z*	*P*
		*P*_25_	*P*_75_		
Outpatients	5000.00	1000.00	10000.00	−5.610	<0.001
Inpatients	8000.00	3000.00	10000.00		

### 3.5 Factors associated with economic burden among outpatients

Univariate analyses indicated that outpatient economic burden differed significantly according to age, occupation, annual *per capita* household income, payment method, antibiotic use, and disease duration (*p* < 0.05). Variables such as sex and the presence of chronic diseases were not significantly associated with any cost category (*p* > 0.05, Supplementary Table S1). GLM analyses showed that participants aged 6—14, 15—59, and ≥60 years had lower total costs than those aged 0—5 years (*p* < 0.05), with the 15—59 and ≥60 groups also showing lower indirect medical costs (*p* < 0.05). Higher household *per capita* income was associated with increased costs in a dose–response manner (*p* < 0.001). Patients with combined insurance and out-of-pocket payment incurred higher indirect medical costs (*p* < 0.05). Patients without antibiotic use had higher direct and total costs, whereas those without chronic diseases had lower costs (*p* < 0.05). Longer disease duration was positively associated with increases in all cost categories (*p* < 0.05). No differences were observed by sex or occupation (*p* > 0.05, Table 5).

**Table 5:** GLM analysis of factors influencing economic burden among outpatients (*n* = 372).

Variables	Direct medical costs				Indirect medical costs				Total costs			
	*β*	*P*	Exp(β)	95%CI	*β*	*P*	Exp(β)	95%CI	*β*	*P*	Exp(β)	95%CI
Gender												
Male	Ref	–	Ref	–	Ref	–	Ref	–	Ref	–	Ref	–
Female	−0.029	0.767	0.971	0.612–1.487	0.201	0.419	1.223	0.752–2.098	0.108	0.702	1.114	0.651–1.782
Age												
0–5 years	Ref	–	Ref	–	Ref	–	Ref	–	Ref	–	Ref	–
6–14 years	−0.173	0.054	0.841	0.570–1.123	−0.184	0.089	0.832	0.593–1.224	−0.196	0.029	0.822	0.559–0.967
15–59 years	−0.693	0.008	0.500	0.320–0.771	−0.256	0.023	0.774	0.513–0.942	−0.393	0.010	0.675	0.420–0.895
≥60 years	−0.158	0.069	0.854	0.523–1.189	−0.414	0.019	0.661	0.410–0.855	−0.247	0.020	0.781	0.410–0.918
Occupation												
Worker and Farmers	Ref	–	Ref	–	Ref	–	Ref	–	Ref	–	Ref	–
Students	−0.040	0.632	0.961	0.589–3.798	−0.082	0.512	0.921	0.389–3.782	−0.091	0.230	0.913	0.401–3.632
Scattered Children and Kindergarten Children	0.220	0.255	1.246	0.631–2.412	0.138	0.321	1.148	0.537–2.323	0.280	0.195	1.323	0.677–4.021
Others	0.034	0.544	1.035	0.489–4.123	0.081	0.488	1.084	0.367–3.781	0.049	0.234	1.050	0.610–3.752
*Per capita* household income												
¥0–50,000	Ref	–	Ref	–	Ref	–	Ref	–	Ref	–	Ref	–
¥50,001–100,000	0.297	0.055	1.345	0.907–2.829	0.270	0.499	1.310	0.698–1.792	0.327	0.045	1.388	1.036–2.827
¥100,001–150,000	0.606	0.020	1.833	1.372–3.226	0.568	0.388	1.765	0.699–2.267	0.611	0.020	1.842	1.225–3.338
≥¥150,001	0.854	0.001	2.349	1.600–4.699	0.751	0.015	2.118	1.221–4.378	0.904	<0.001	2.469	1.431–4.562
Payment method												
Medical Insurance	Ref	–	Ref	–	Ref	–	Ref	–	Ref	–	Ref	–
Out-of-pocket	−0.401	0.489	0.670	0.228–2.098	−0.520	0.467	0.595	0.201–2.199	−0.443	0.389	0.642	0.124–1.427
Medical insurance + out-of-pocket	0.404	0.076	1.498	0.952–2.499	0.521	0.032	1.684	1.088–3.329	0.477	0.070	1.611	0.867–3.118
Antibiotics												
Yes	Ref	–	Ref	–	Ref	–	Ref	–	Ref	–	Ref	–
No	0.150	0.023	1.162	1.055–1.347	0.069	0.786	1.071	0.602–1.885	0.168	0.045	1.183	1.020–1.882
Chronic disease												
Yes	Ref	–	Ref	–	Ref	–	Ref	–	Ref	–	Ref	–
No	−0.702	0.035	0.496	0.156–0.878	−1.155	0.080	0.315	0.084–1.142	−0.699	0.020	0.497	0.268–0.839
Duration of illness												
0–5 days	Ref	–	Ref	–	Ref	–	Ref	–	Ref	–	Ref	–
6–10 days	0.243	0.091	1.275	0.823–1.956	0.634	0.401	1.885	0.658–3.239	0.297	0.208	1.321	0.845–2.579
11–15 days	0.605	0.001	1.829	1.554–2.316	0.744	0.047	2.104	1.018–4.235	0.673	0.021	1.960	1.278–3.864
16–20 days	0.711	<0.001	2.035	1.823–4.125	0.837	0.005	2.310	1.551–6.458	0.810	0.001	2.246	1.624–5.934
≥21 days	0.734	<0.001	2.084	1.993–4.298	1.037	<0.001	2.821	1.240–7.446	0.920	<0.001	2.509	1.865–6.261

### 3.6 Univariate analysis of factors associated with inpatients economic burden

Among inpatients, age group, occupation, payment method, disease duration, antibiotic use, and the presence of chronic diseases were significantly associated with variations in economic burden (*p* < 0.05). Annual household income showed significant associations with direct medical costs, total costs, and intangible costs. Sex was not significantly associated with any cost category (*p* > 0.05, Supplementary Table S2). GLM analyses showed that older age groups (6—14, 15—59, and ≥60 years) had lower direct medical and total costs than those aged 0—5 years (*p* < 0.05). Students and kindergarten/scattered children incurred higher direct and total costs (*p* < 0.05). Higher household *per capita* income was associated with increased costs in a dose–response manner (*p* < 0.001). No significant differences were observed by sex or payment method (*p* > 0.05). Patients without antibiotic use had higher direct and total costs, whereas those without chronic diseases had lower costs (*p* < 0.05). All cost components increased progressively with longer disease duration (*p* < 0.05, Table 6). Model diagnostics indicated acceptable goodness-of-fit of the GLM models.

**Table 6:** GLM analysis of factors influencing economic burden among inpatients (*n* = 210).

Variables	Direct medical costs				Indirect medical costs				Total costs			
	*β*	*P*	Exp(β)	95%CI	*β*	*P*	Exp(β)	95%CI	*β*	*P*	Exp(β)	95%CI
Gender												
Male	Ref	–	Ref	–	Ref	–	Ref	–	Ref	–	Ref	–
Female	−0.080	0.101	0.923	0.645–1.226	0.121	0.523	1.129	0.620–2.212	−0.102	0.884	0.903	0.612–1.683
Age												
0–5 years	Ref	–	Ref	–	Ref	–	Ref	–	Ref	–	Ref	–
6–14 years	−0.177	0.046	0.838	0.520–0.964	−0.118	0.414	0.889	0.535–1.427	−0.099	0.310	0.906	0.572–1.308
15–59 years	−0.557	<0.001	0.573	0.333–0.834	−0.086	0.426	0.918	0.531–1.533	−0.323	0.020	0.724	0.482–0.971
≥60 years	−0.331	0.010	0.718	0.439–0.913	−0.114	0.422	0.892	0.540–1.561	−0.130	0.102	0.878	0.513–1.169
Occupation												
Worker and Farmers	Ref	–	Ref	–	Ref	–	Ref	–	Ref	–	Ref	–
Students	0.300	0.002	1.350	1.196–3.298	−0.048	0.541	0.953	0.689–2.629	0.240	0.020	1.271	1.052–2.641
Scattered Children and Kindergarten Children	0.528	<0.001	1.695	1.269–7.032	0.042	0.456	1.043	0.518–3.209	0.338	<0.001	1.402	1.071–6.936
Others	0.119	0.224	1.126	0.671–2.935	−0.046	0.521	0.955	0.456–2.834	0.100	0.427	1.105	0.599–2.607
*Per capita* household income												
¥0–50,000	Ref	–	Ref	–	Ref	–	Ref	–	Ref	–	Ref	–
¥50,001–100,000	0.477	0.027	1.612	1.289–3.687	0.048	0.672	1.049	0.400–1.802	0.238	0.060	1.269	0.953–5.628
¥100,001–150,000	0.648	0.004	1.912	1.445–4.209	0.198	0.383	1.219	0.611–1.818	0.647	0.022	1.910	1.334–6.104
≥¥150,001	0.782	<0.001	2.186	1.423–11.341	0.535	0.087	1.708	0.860–3.139	0.762	<0.001	2.143	1.462–9.390
Payment Method												
Medical Insurance	Ref	–	Ref	–	Ref	–	Ref	–	Ref	–	Ref	–
Out-of-pocket	−0.074	0.534	0.929	0.612–4.534	0.120	0.234	1.128	0.520–4.454	0.082	0.577	1.085	0.753–3.163
Medical insurance + out-of-pocket	0.063	0.432	1.065	0.888–6.467	0.145	0.188	1.156	0.757–4.107	0.094	0.334	1.098	0.664–5.821
Antibiotics												
Yes	Ref	–	Ref	–	Ref	–	Ref	–	Ref	–	Ref	–
No	0.236	0.025	1.266	1.046–1.823	−0.051	0.664	0.950	0.443–2.321	0.204	0.030	1.226	1.052–1.636
Chronic disease												
Yes	Ref	–	Ref	–	Ref	–	Ref	–	Ref	–	Ref	–
No	−0.326	0.040	0.722	0.491–0.943	−0.323	0.103	0.710	0.260–1.422	−0.305	0.032	0.737	0.456–0.961
Duration of illness												
0–5 days	Ref	–	Ref	–	Ref	–	Ref	–	Ref	–	Ref	–
6–10 days	0.322	0.020	1.380	1.164–2.712	0.252	0.051	1.287	0.981–2.823	0.216	0.020	1.241	1.180–2.709
11–15 days	0.684	0.001	1.982	1.215–3.235	0.308	0.028	1.361	1.159–2.935	0.461	0.002	1.586	1.245–3.212
16–20 days	0.751	<0.001	2.120	1.366–3.754	0.578	<0.001	1.782	1.205–4.123	0.618	<0.001	1.855	1.466–4.172
≥21 days	0.807	<0.001	2.240	1.531–4.324	0.843	<0.001	2.323	1.402–4.474	0.899	<0.001	2.433	1.592–4.458

## 4 Discussion

This multicenter stratified sampling study systematically quantified the economic burden of MP infection and its determinants among outpatient and inpatient populations in Jinhua City. The findings indicate that MP infection imposes considerable direct, indirect, and intangible economic burdens on affected households, with notable differences in cost structures across healthcare settings. These results suggest that the economic impact of MP infection extends beyond clinical consequences and represents an important public health and health-financing challenge, particularly among pediatric populations.

Our study population contained a relatively large proportion of pediatric patients, which is consistent with the well-established epidemiological pattern that MP infection predominantly affects children and adolescents. Previous epidemiological studies have similarly reported higher infection rates and healthcare utilization among pediatric populations, reflecting the increased susceptibility of children to MP infection and their greater likelihood of seeking medical care (11, 17). Because pediatric patients often require parental accompaniment during treatment and recovery, the illness burden frequently extends beyond the patient to the entire household.

A pronounced disparity in economic burden was observed between outpatients and inpatients. The total *per capita* cost among inpatients was approximately 7.7 times higher than that of outpatients, a magnitude comparable to previous reports on respiratory infections in China (19, 20). Such differences likely reflect greater clinical severity among hospitalized patients, longer length of hospital stay, increased diagnostic intensity, and the need for more aggressive therapeutic interventions. In terms of cost composition, outpatient expenditure was characterized by a relatively balanced contribution of direct and indirect medical costs, whereas inpatient expenditure was predominantly driven by direct medical costs. Among outpatients, productivity loss and other indirect expenditures such as transportation and additional nutritional costs accounted for a large proportion of total costs. This pattern is closely related to the high proportion of pediatric cases in the present study. Children often require continuous parental care during illness, leading to work absenteeism and consequent household income loss. Therefore, outpatient economic burden was largely driven by time-related productivity losses, whereas inpatient burden was primarily driven by intensive medical interventions and hospital-based expenditures. In pediatric patients, caregiver-related productivity loss may further amplify the indirect economic burden borne by households. Similar patterns of indirect-cost dominance in pediatric respiratory infections have been reported in previous studies (21), highlighting the hidden but substantial socioeconomic impact borne by families.

In contrast, inpatient economic burden was dominated by direct medical costs, accounting for nearly four-fifths of total expenditure. Diagnostic testing and comprehensive service fees constituted the largest cost components, reflecting the clinical complexity of inpatients MP infection, which frequently necessitates extensive diagnostic evaluation, prolonged treatment, and continuous nursing care (22). Previous international surveillance reports have likewise shown that direct medical costs comprise the majority of expenditure among inpatient MP patients (23, 24), suggesting a broadly consistent cost structure for severe MP infection across healthcare systems.

Regarding absolute cost levels, the *per capita* direct medical expenditure for outpatients with MP infection in this study was relatively lower than estimates for pertussis and other acute respiratory infections in previous Chinese studies (25). This difference may reflect recent improvements in medical insurance policies, reductions in diagnostic test pricing, and strengthened antimicrobial stewardship in China. Conversely, inpatient direct medical costs for MP infection in the present study were higher than those reported for bronchiolitis, pneumonia, and other respiratory infections in other Chinese hospitals (26). This increase may be attributed to prolonged treatment courses resulting from rising antimicrobial resistance and the expanded use of advanced diagnostic technologies, such as nucleic acid testing. Given the growing prevalence of macrolide-resistant MP strains in the Asia-Pacific region (27), resistant infections frequently necessitate higher-tier or combination antimicrobial therapies, thereby increasing treatment costs and amplifying the economic burden relative to other common respiratory pathogens.

Medical insurance coverage partially mitigated the economic burden but did so unevenly across care settings. In this study, hospitalized patients benefited from higher reimbursement rates than outpatients, which is consistent with China’s health insurance policy framework that traditionally prioritizes inpatient coverage (28). However, reimbursement levels in both settings remain lower than those reported in developed countries such as Germany (29). This gap suggests potential room for policy optimization, particularly for outpatient respiratory infections, where families—especially those with pediatric patients—bear considerable indirect and out-of-pocket costs.

The intangible economic burden represents an important yet often overlooked component of the total disease burden (30). This study provides a systematic evaluation of intangible burden using the WTP method, representing a methodological strength of the study. The intangible burden among inpatients was markedly higher than that among outpatients, suggesting that increasing disease severity is accompanied by greater psychological distress and perceived health loss (31). This phenomenon may be particularly pronounced in pediatric cases, where parental anxiety regarding disease progression, potential complications, and long-term health outcomes may substantially amplify psychological stress (32). Furthermore, the wide interquartile range observed in WTP estimates indicates substantial heterogeneity in perceived disease burden among patients and their families. Such variability may reflect differences in disease severity, socioeconomic status, and individual perceptions of health-related quality of life. Although the WTP approach provides a useful estimate of intangible burden, the results may be influenced by respondents’ income levels and subjective perceptions of suffering. Individuals with higher income may report higher WTP values regardless of actual disease severity. This may lead to overestimation of intangible costs. In addition, variations in respondents’ understanding of “complete avoidance of suffering” may introduce measurement bias. Future studies could consider incorporating standardized health utility instruments, such as the EQ-5D, to complement and validate WTP-based estimates.

Analysis of influencing factors identified several key determinants of economic burden. Prolonged disease duration was the most consistent factor, showing a clear dose–response relationship with direct, indirect, and total costs across both outpatient and inpatient groups. Interestingly, patients who did not receive antibiotic treatment incurred higher direct and total costs. This counterintuitive finding may reflect delayed initiation of appropriate antimicrobial therapy, resulting in prolonged disease duration, additional diagnostic testing, and increased healthcare utilization before definitive treatment was established. This finding reinforces the critical importance of early diagnosis and timely intervention in mitigating economic burden (33). Comorbidities also markedly increased inpatient costs, reflecting the greater disease severity and the need for more intensive medical care in patients with underlying conditions (34). Furthermore, higher household income was associated with both higher medical expenditures and higher intangible costs. Higher-income households may have greater access to healthcare services and a higher likelihood of utilizing diagnostic and therapeutic resources, leading to increased observed medical expenditures. In addition, willingness to pay for relief from physical and psychological suffering generally increases with economic capacity (35).

Several limitations should be acknowledged. First, the study population was recruited from secondary and tertiary hospitals, which may have led to an overestimation of the overall economic burden because milder cases managed in primary care or community settings were not included. Second, this study did not systematically assess co-infection with other respiratory pathogens. Polymicrobial infections are common in lower respiratory tract infections, particularly among adults and older adults individuals with underlying conditions. Therefore, part of the observed economic burden may be attributable to co-pathogens rather than MP infection alone. Third, indirect cost estimation relied partly on self-reported information, which may be subject to recall bias despite efforts to minimize this through timely interviews and verification with medical records. Fourth, intangible costs were assessed using the WTP approach, which may be influenced by respondents’ economic capacity and potential hypothetical bias. Fifth, although generalized linear models were applied to better accommodate the skewed distribution of cost data, residual confounding cannot be excluded due to the observational study design. In addition, although the overall sample size met the minimum requirement, the inpatient subgroup had a relatively smaller sample size, which may have limited statistical power for detecting small effect sizes in multivariable analyses. Therefore, findings for inpatients should be interpreted with caution. Future studies should incorporate multiplex pathogen testing to distinguish mono-infection from co-infection and adopt longitudinal designs to better assess causal relationships and temporal changes in economic burden. In addition, stratified analyses according to antimicrobial resistance status may further improve understanding of the determinants of economic burden.

In conclusion, MP infection imposes a substantial economic burden on patients in China, particularly among children, with burden structure varying markedly by healthcare setting. Disease duration, comorbidities, healthcare utilization intensity, and medical insurance coverage are key determinants of economic burden. These findings may provide useful evidence for policymakers considering strategies to reduce the economic burden of MP infection, such as improving outpatient reimbursement mechanisms and supporting future vaccine development. Such measures are essential for reducing the economic burden of MP infection at both the household and population levels.

## Data Availability

The original contributions presented in the study are included in the article/Supplementary material, further inquiries can be directed to the corresponding authors.
