# Supplementary material for: Economic burden of Mycoplasma pneumoniae infection in Jinhua City, China: a multicenter cross-sectional study
Source: Front Public Health. 2026 Jun 1;14:1835645. doi: 10.3389/fpubh.2026.1835645 (PMC13265536; doi:10.3389/fpubh.2026.1835645)
Supplement: Supplementary file 1 [file Supplementary_file_1.DOCX]

Supplementary Material

# Supplementary Tables

Supplementary Tables 1. Univariate Analysis of Factors Influencing the Economic Burden of Outpatients (n = 372, ¥).

Supplementary Tables 2. Univariate Analysis of Factors Influencing the Economic Burden of Inpatients (n = 210, ¥)

# Supplementary File

Mycoplasma pneumoniae Infection Economic Burden Survey Questionnaire (English version)

Tables S1. Univariate Analysis of Factors Influencing the Economic Burden of Outpatients(n = 372, ¥)

| Variables | Direct Medical Costs | Indirect Medical Costs | Total Costs | Intangible Costs |
| --- | --- | --- | --- | --- |
| Gender |  |  |  |  |
| Male | 341.60(199.25,490.60) | 188.10(17.50,381.20) | 547.80(348.35,1065.30) | 5000.00(1000.00,10000.00) |
| Female | 316.20(193.40,480.40) | 188.80(21.50,386.20) | 551.60(351.80,1064.50) | 3000.00(1000.00,8000.00) |
| *Z* | -1.455 | -0.507 | -0.006 | -0.094 |
| *P* | 0.146 | 0.612 | 0.995 | 0.925 |
| Age |  |  |  |  |
| 0-5 years | 453.50(199.25,644.30) | 372.20(188.60,1105.10) | 830.50(556.20,1645.35) | 5000.00(3000.00,10000.00) |
| 6-14 years | 309.00(180.60,478.58) | 198.10(187.10,747.40) | 643.80(374.13,1260.65) | 2000.00(1000.00,10000.00) |
| 15-59 years | 302.50(208.85,457.00) | 15.00(5.00,185.60) | 390.90(251.80,611.85) | 5000.00(1000.00,9000.00) |
| ≥60 years | 377.45(218.58,519.53) | 187.10(9.38,371.20) | 565.80(364.48,999.50) | 1000.00(1000.00,5000.00) |
| *H* | 6.234 | 109.539 | 55.188 | 13.781 |
| *P* | 0.101 | <0.001 | <0.001 | 0.003 |
| Occupation |  |  |  |  |
| Worker and Farmers | 237.10(188.48,476.50) | 10.00(1.00,149.50) | 267.65(232.70,480.75) | 1000.00(1000.00,8750.00) |
| Students | 306.50(182.40,444.30) | 192.10(187.10,739.90) | 622.70(370.50,1123.10) | 5000.00(3000.00,10000.00) |
| Scattered Children and Kindergarten Children | 388.30(194.73,649.08) | 370.70(188.10,1109.73) | 809.15(516.38,1672.38) | 5000.00(1000.00,10000.00) |
| Others | 368.15(215.60,479.03) | 48.75(9.00,188.50) | 479.40(264.95,719.78) | 3000.00(1000.00,8000.00) |
| *H* | 5.022 | 104.984 | 43.664 | 30.556 |
| *P* | 0.657 | <0.001 | 0.007 | <0.001 |
| Per capita household income |  |  |  |  |
| ¥0-50000 | 239.00(84.40,469.50) | 188.10(6.50,551.80) | 412.30(263.80,1327.30) | 1000.00(1000.00,5000.00) |
| ¥50001-100000 | 245.35(185.40,433.43) | 188.10(36.25,381.20) | 434.35(260.65,683.90) | 5000.00(1000.00,10000.00) |
| ¥100,001-150,000 | 269.80(166.05,405.50) | 187.60(40.00,203.10) | 507.90(329.35,922.70) | 9000.00(3000.00,20000.00) |
| ≥¥150001 | 393.85(246.00,607.00) | 198.10(40.00,1113.60) | 624.25(391.90,1213.55) | 10000.00(3000.00,100000.00) |
| *H* | 25.211 | 2.769 | 10.137 | 55.505 |
| *P* | <0.001 | 0.429 | 0.017 | <0.001 |
| Payment Method |  |  |  |  |
| Medical Insurance | 312.50(193.08,469.58) | 187.10(15.00,201.60) | 484.95(340.10,794.78) | 1000.00(1000.00,5000.00) |
| Out-of-pocket | 299.45(184.83,544.43) | 185.60(21.25,330.18) | 421.00(287.70,925.45) | 5000.00(1000.00,5000.00) |
| Medical insurance + out-of-pocket | 349.45(211.15,558.35) | 330.60(60.00,1111.48) | 768.45(397.46,1638.35) | 5000.00(1000.00,10000.00) |
| *H* | 1.571 | 19.506 | 17.175 | 27.373 |
| *P* | 0.456 | <0.001 | <0.001 | <0.001 |
| Antibiotics |  |  |  |  |
| Yes | 258.50(156.40,469.50) | 188.10(22.50,381.20) | 510.30(331.60,1064.50) | 5000.00(1000.00,10000.00) |
| No | 337.70(220.00,490.95) | 188.10(20.00,564.30) | 552.40(356.30,1066.95) | 5000.00(1000.00,5000.00) |
| *Z* | -2.470 | -0.256 | -0.518 | -0.771 |
| *P* | 0.014 | 0.789 | 0.604 | 0.441 |
| Chronic disease |  |  |  |  |
| Yes | 400.55(241.38,608.98) | 186.60(15.00,373.08) | 621.85(460.40,1038.13) | 3000.00(1000.00,7250.00) |
| No | 317.00(192.10,480.25) | 188.10(22.38,381.20) | 536.05(341.23,1069.30) | 5000.00(1000.00,10000.00) |
| *Z* | -1.680 | -0.555 | -1.020 | -0.856 |
| *P* | 0.093 | 0.579 | 0.308 | 0.392 |
| Duration of illness |  |  |  |  |
| 0-5 days | 251.00(137.20,385.70) | 188.10(47.50,381.20) | 489.00(294.50,808.00) | 1000.00(1000.00,5000.00) |
| 6-10 days | 327.60(211.20,480.40) | 188.10(15.00,381.20) | 540.40(336.10,1250.20) | 5000.00(1000.00,10000.00) |
| 11-15 days | 395.00(252.58,586.20) | 198.60(98.52,753.15) | 569.60(396.88,773.75) | 5000.00(1000.00,10000.00) |
| 16-20 days | 467.05(189.75,963.65) | 186.60(43.75,384.95) | 737.30(438.20,1456.35) | 5000.00(5000.00,10000.00) |
| ≥21 days | 442.35(243.13,576.15) | 173.36(5.75,195.85) | 764.00(359.95,1402.85) | 5000.00(5000.00,10000.00) |
| *H* | 26.980 | 9.356 | 12.177 | 19.999 |
| *P* | <0.001 | 0.053 | 0.016 | <0.001 |

Tables S2 Univariate Analysis of Factors Influencing the Economic Burden of Inpatients(n = 210, ¥)

| Variables | Direct Medical  Costs | Indirect Medical Costs | Total Costs | Intangible Costs |
| --- | --- | --- | --- | --- |
| Gender |  |  |  |  |
| Male | 4175.20(2879.30,6550.70) | 1219.50(598.30,1933.60) | 5543.40(3850.70,7831.30) | 5000.00(3000.00,10000.00) |
| Female | 4020.60(3172.80,6422.00) | 1182.40(605.00,1847.90) | 5634.50(4087.00,7770.00) | 8000.00(3000.00,15000.00) |
| *Z* | -0.070 | -0.007 | -0.084 | -1.307 |
| *P* | 0.944 | 0.995 | 0.933 | 0.191 |
| Age |  |  |  |  |
| 0-5 years | 3985.70(2771.75,4954.10) | 1746.70(1135.50,2221.35) | 5685.00(4029.80,8114.65) | 5000.00(1000.00,10000.00) |
| 6-14 years | 3188.00(2450.70,4063.70) | 1125.10(688.73,1747.75) | 4473.75(3417.58,6106.78) | 5000.00(3000.00,10000.00) |
| 15-59 years | 5093.50(3712.30,7200.20) | 1025.50(192.50,1883.60) | 6440.70(4589.80,8465.70) | 8000.00(5000.00,15000.00) |
| ≥60 years | 6747.55(3939.27,8713.65) | 1192.30(655.55,2523.15) | 7965.70(5505.15,10663.45) | 9000.00(4000.00,27000.00) |
| *H* | 45.583 | 12.163 | 32.000 | 5.139 |
| *P* | <0.001 | 0.007 | <0.001 | 0.162 |
| Occupation |  |  |  |  |
| Worker and Farmers | 3775.60(3316.25,4921.70) | 815.00(152.75,2828.75) | 4473.75(3525.10,6141.70) | 1000.00(1000.00,7500.00) |
| Students | 3282.55(2499.83,4194.55) | 1194.35(664.05,1784.98) | 5631.00(3723.75,7410.50) | 8000.00(3000.00,10000.00) |
| Scattered Children and Kindergarten Children | 3900.30(2771.75,4614.71) | 1607.70(935.65,2069.20) | 5898.80(4703.75,7998.33) | 9000.00(3500.00,15000.00) |
| Others | 5054.35(3606.18,6878.28) | 791.15(78.13,1600.27) | 5548.90(3815.45,6950.15) | 5000.00(1000.00,9000.00) |
| *H* | 51.728 | 10.097 | 36.062 | 14.535 |
| *P* | <0.001 | 0.183 | <0.001 | 0.042 |
| Per capita household income |  |  |  |  |
| ¥0-50000 | 3881.30(2774.05,5431.75) | 1165.10(681.95,1727.95) | 5402.70(3681.25,7119.75) | 5000.00(1000.00,9000.00) |
| ¥50001-100000 | 4267.35(3411.00,6529.43) | 1191.85(605.00,1982.03) | 5912.40(4484.95,7984.45) | 8000.00(5000.00,10000.00) |
| ¥100,001-150,000 | 4249.20(3259.80,6575.50) | 1533.60(570.60,2033.10) | 6124.00(4253.20,7855.60) | 15000.00(10000.00,20000.00) |
| ≥¥150001 | 5282.80(2872.73,7009.68) | 895.85(307.50,1657.35) | 5868.65(4295.58,8499.45) | 20000.00(9500.00,62500.00) |
| *H* | 2.804 | 1.366 | 3.126 | 42.853 |
| *P* | 0.423 | 0.713 | 0.373 | <0.001 |
| Payment Method |  |  |  |  |
| Medical Insurance | 3227.40(2324.05,4186.30) | 1141.60(892.40,1535.20) | 4168.90(3339.85,5659.75) | 3000.00(1000.00,10000.00) |
| Out-of-pocket | 5459.60(3478.70,7177.50) | 1310.50(20.00,1985.70) | 6967.40(3498.70,8527.90) | 8000.00(3000.00,10000.00) |
| Medical insurance + out-of-pocket | 4710.10(3496.73,7183.18) | 1275.50(513.75,1990.18) | 6247.90(4593.48,8536.73) | 8000.00(5000.00,15000.00) |
| *H* | 35.724 | 0.499 | 34.204 | 12.100 |
| *P* | <0.001 | 0.779 | <0.001 | 0.002 |
| Antibiotics |  |  |  |  |
| Yes | 3701.90(2758.20,4754.20) | 1182.40(854.09,1746.70) | 4864.50(3748.50,6363.50) | 3000.00(1000.00,10000.00) |
| No | 4333.10(3227.40,6962.70) | 1219.50(554.30,1975.50) | 5794.50(4299.40,8340.20) | 8000.00(5000.00,15000.00) |
| *Z* | -2.796 | -0.151 | -2.607 | -3.959 |
| *P* | 0.005 | 0.880 | 0.009 | <0.001 |
| Chronic disease |  |  |  |  |
| Yes | 6616.50(4554.20,8205.53) | 1224.05(605.00,1885.63) | 7744.15(5507.23,10046.53) | 8000.00(5000.00,15000.00) |
| No | 3806.70(2845.28,5679.35) | 1148.70(464.83,1826.33) | 5306.45(3753.18,6991.75) | 8000.00(3000.00,10000.00) |
| *Z* | -4.767 | -0.435 | -4.104 | 0.921 |
| *P* | <0.001 | 0.663 | <0.001 | 0.357 |
| Duration of illness |  |  |  |  |
| 0-5 days | 2766.80(2047.10,4412.60) | 892.40(554.30,1092.50) | 3748.90(2793.40,4984.10) | 5000.00(3000.00,10000.00) |
| 6-10 days | 4198.10(3402.50,6637.90) | 1291.70(520.00,1796.70) | 5314.50(3708.80,7886.80) | 8000.00(3000.00,10000.00) |
| 11-15 days | 4014.20(3033.00,6550.70) | 1762.65(882.40,2291.00) | 5748.20(4284.90,8434.30) | 9000.00(5000.00,15000.00) |
| 16-20 days | 4249.20(2726.45,7280.40) | 1883.60(1053.80,2576.65) | 6350.85(4471.90,8705.15) | 8000.00(3000.00,15000.00) |
| ≥21 days | 5382.35(3325.83,8550.03) | 1938.60(1126.28,2841.13) | 6997.90(5208.40,11327.15) | 20000.00(10000.00,35000.00) |
| *H* | 23.452 | 25.162 | 31.729 | 13.089 |
| *P* | <0.001 | <0.001 | <0.001 | 0.011 |

Supplementary File

**Mycoplasma pneumoniae Infection Economic Burden Survey Questionnaire (English version)**

Case ID: □□□

Dear participant,

Thank you very much for taking the time to participate in this survey. This study is commissioned by the Jinhua Municipal Health Authority to assess the economic burden of Mycoplasma pneumoniae infection. All information collected will be kept strictly confidential and used for research purposes only!

Respondent:______ 1）Patient 2）Family member;

Patient type:______ 1）Outpatient 2）Inpatient

Survey date: ____ / ____ / 202_

Survey institution: Investigator:

| Section I. General Information |
| --- |
| 1. Patient name:_________________ Telephone number:__________________________ |
| 2. Sex:___ 1）Male 2）Female |
| 3. Date of birth: ____ / ____ / ______  Age group: （1）0-5 years（2）6-14 years（3）15-59 years（4）≥60 years |
| 4. Ethnicity: ___ 1）Han 2）Others (please specify): __________ |
| 5. Current residence: ______ City ______ County/District ______ Town/Street ______ Village |
| 6. Educational level: （1）No formal education（2）Primary school（3）Junior high school（4）enior high/technical school（5）College/University（6）Master’s degree or above |
| 7. Marital status: （1）Married （2）Widowed （3）Divorced （4）Single |
| 8. Occupation: （1）Worker (including migrant worker)（2）Farmer/Fisherman/Herder（3）Public administration/institution staff（4）Professional/technical staff（5）Commercial/service personnel（6）Retired（7）Unemployed （8）Student（9）Scattered Children（10）Kindergarten Children （11）Others __________ |
| 9. Number of family members: ______ persons |
| 10. Annual household income: ______ ×10,000 RMB |
| 11. Medical payment method (multiple choice allowed):（1）Urban-rural resident basic medical insurance（2）Urban employee basic medical insurance（3）New rural cooperative medical scheme（4）Retired cadre medical insurance（5）Self-paid（6）Commercial insurance（7）Others__________ |
| 12. History of chronic disease or cancer: （1）Yes （2）No  If yes: （1）Chronic bronchitis（2）Emphysema（3）Hypertension（4）Coronary heart disease（5）Diabetes （6）Others __________ |

| Section II. Outpatient/Emergency Treatment | | | | |
| --- | --- | --- | --- | --- |
| 1 | Did you receive outpatient/emergency treatment? （1）Yes（2）No | | | |
| 2 | Date of onset: ____ / ____ / ______ Total illness duration: ______ days | | | |
| 3 | Antibiotics used?____ （1）Yes（2）No；  If yes, names: ___________________  Start date: ____ / ____ / ______ | | | |
| 4 | During this illness and treatment, the total number of outpatient visits was: times (multiple visits to one hospital are combined into one). | | | |
| 5 | Outpatient treatment history | First time | Second time | Third time |
| 6 | Hospital Name |  |  |  |
| 7 | Total medical expenses (RMB)  * Note: Respondents were first asked to recall previous medical expenses; if they could not recall, they were given a hint: 1) <100; 2) 100-299; 3) 300-499; 4) 500-799；5) 800-999; 6) 1000-1999; 7)2000-2999; 8) ≥3000 | RMB  If you cannot recall, please fill in the amount option on the left. | RMB  If you cannot recall, please fill in the amount option on the left. | RMB  If you cannot recall, please fill in the amount option on the left. |
|  | Of which: Comprehensive Services |  |  |  |
|  | Diagnostic and Laboratory Services |  |  |  |
|  | Therapeutic |  |  |  |
|  | Pharmaceuticals |  |  |  |
|  | Consumables |  |  |  |
|  | Of which: Out-of-pocket expenses (RMB)  *Note: Respondents were first asked to recall previous medical expenses; if they could not recall, they were given a hint: 1) <100; 2) 100-299; 3) 300-499; 4) 500-799；5) 800-999; 6) 1000-1999; 7) 2000-2999; 8) ≥3000 | RMB  If you cannot recall, please fill in the amount option on the left. | RMB  If you cannot recall, please fill in the amount option on the left. | RMB  If you cannot recall, please fill in the amount option on the left. |
| 8 | Did you purchase any medication from a pharmacy? If so, how much did it cost? (RMB). Enter 0 if not.  (Interviewer's note: This includes all medication costs related to Mycoplasma pneumoniae infection purchased by the patient and their family.) | RMB  If they cannot recall it, provide hints:  1) <30; 2) 30-49; 3) 50-99; 4)100-199; 5) 200-499;  6)500-999; 7) ≥1000. | | |
|  | Out-of-pocket | RMB  If they cannot recall it, provide hints:  1) <30；2) 30-49；3) 50-99；4)100-199；5) 200-499；  6)500-999；7) ≥1000。 | | |
| 9 | Number of accompanying family members |  |  |  |
|  | Average days of accompaniment per person |  |  |  |
| 10 | Transportation costs for patient and caregivers (round trip): ______ RMB | RMB  If they cannot recall it, provide hints:  1) <10  2) 10-29  3) 30-49  4) 50-99  5)100-199  6) ≥200  7）If you are driving a private car and the cost cannot be estimated, please provide the total distance (round trip) in kilometers . Private vehicle types: a. Cars, b. Motorcycles, c. Bicycles, d. Others | RMB  If they cannot recall it, provide hints:  1) <10  2) 10-29  3) 30-49  4) 50-99  5)100-199  6) ≥200  7）If you are driving a private car and the cost cannot be estimated, please provide the total distance (round trip) in kilometers . Private vehicle types: a. Cars, b. Motorcycles, c. Bicycles, d. Others | RMB  If they cannot recall it, provide hints:  1) <10  2) 10-29  3) 30-49  4) 50-99  5)100-199  6) ≥200  7）If you are driving a private car and the cost cannot be estimated, please provide the total distance (round trip) in kilometers . Private vehicle types: a. Cars, b. Motorcycles, c. Bicycles, d. Others |

| Section III. Inpatient Treatment | | | | |
| --- | --- | --- | --- | --- |
| 1 | Hospitalization? （1）Yes（2）No. If not, skip to Section VI. | | | |
| 2 | Number of hospitalizations: ________ | | | |
| 3 | Hospitalization treatment history | First time | Second time | Third time |
| 4 | Hospital name |  |  |  |
|  | Department |  |  |  |
| 5 | Length of stay(days) |  |  |  |
| 6 | Discharge diagnosis |  |  |  |
| 7 | Severe case: 1)Yes 2)No |  |  |  |
| 8 | Total cost of this hospitalization (RMB)  *Note: Costs for the most recent survey must be obtained through medical record inquiries. For previous medical expenses, respondents should first recall them; if they cannot recall, prompts will be provided:1) <3000; 2) 3000-3999;  3) 4000-4999; 4) 5000-9999;  5) 10000-19999; 6) 20000-29999;  7) ≥30000 | RMB  If you cannot recall, please fill in the amount option on the left. | RMB  If you cannot recall, please fill in the amount option on the left. | RMB  If you cannot recall, please fill in the amount option on the left. |
|  | Of which: Comprehensive Services |  |  |  |
|  | Diagnostic and Laboratory Services |  |  |  |
|  | Therapeutic |  |  |  |
|  | Pharmaceuticals |  |  |  |
|  | Consumables |  |  |  |
|  | Of which: Out-of-pocket expenses (RMB)  *Note: Respondents were first asked to recall previous medical expenses; if they could not recall, they were given a hint:1) <3000; 2) 3000-3999;  3) 4000-4999; 4) 5000-9999;  5) 10000-19999; 6) 20000-29999;  7) ≥30000 | RMB  If you cannot recall, please fill in the amount option on the left. | RMB  If you cannot recall, please fill in the amount option on the left. | RMB  If you cannot recall, please fill in the amount option on the left. |
| 9 | Number of accompanying family members |  |  |  |
|  | Average days of accompaniment per person |  |  |  |
| 10 | Transportation costs for patient and caregivers (round trip): ______ RMB | RMB  If they cannot recall it, provide hints:  1) <10  2) 10-29  3) 30-49  4) 50-99  5)100-199  6) ≥200  7）If you are driving a private car and the cost cannot be estimated, please provide the total distance (round trip) in kilometers. . Private vehicle types: a. Cars, b. Motorcycles, c. Bicycles, d. Others | RMB  If they cannot recall it, provide hints:  1) <10  2) 10-29  3) 30-49  4) 50-99  5)100-199  6) ≥200  7）If you are driving a private car and the cost cannot be estimated, please provide the total distance (round trip) in kilometers. . Private vehicle types: a. Cars, b. Motorcycles, c. Bicycles, d. Others | RMB  If they cannot recall it, provide hints:  1) <10  2) 10-29  3) 30-49  4) 50-99  5)100-199  6) ≥200  7）If you are driving a private car and the cost cannot be estimated, please provide the total distance (round trip) in kilometers. . Private vehicle types: a. Cars, b. Motorcycles, c. Bicycles, d. Others |
| 11 | What is the average daily accommodation cost per accompanying family member during hospitalization? (Enter 0 if none, enter the amount if yes) | RMB/day  If they cannot recall it, provide hints:  1) <100  2) 100-199  3) 200-299  4)300-399  5)400-499  6) ≥500 | RMB/day  If they cannot recall it, provide hints:  1) <100  2) 100-199  3) 200-299  4)300-399  5)400-499  6) ≥500 | RMB/day  If they cannot recall it, provide hints:  1) <100  2) 100-199  3) 200-299  4)300-399  5)400-499  6) ≥500 |
| 12 | What is the average daily food cost per family member accompanying a patient during hospitalization? (Enter 0 if none, enter the amount if applicable) | RMB/day  If they cannot recall it, provide hints:  1) <20  2) 20-49  3) 50-99  4)100-199  5)200-299  6) ≥300 | RMB/day  If they cannot recall it, provide hints:  1) <20  2) 20-49  3) 50-99  4)100-199  5)200-299  6) ≥300 | RMB/day  If they cannot recall it, provide hints:  1) <20  2) 20-49  3) 50-99  4)100-199  5)200-299  6) ≥300 |
| 13 | How much does it cost per day for the patient's nutrition, health supplements, and snacks during hospitalization? (Enter 0 if none, enter the amount if applicable) | RMB/day  If they cannot recall it, provide hints:  1) <20  2) 20-49  3) 50-99  4)100-199  5)200-299  6) ≥300 | RMB/day  If they cannot recall it, provide hints:  1) <20  2) 20-49  3) 50-99  4)100-199  5)200-299  6) ≥300 | RMB/day  If they cannot recall it, provide hints:  1) <20  2) 20-49  3) 50-99  4)100-199  5)200-299  6) ≥300 |
| 14 | Do you require a dedicated caregiver? 1) Yes; 2)No |  |  |  |
|  | How much does it cost to hire a professional caregiver? | RMB  If they cannot recall it, provide hints:  1) <200  2)200-499  3) 500-999  4) 1000-1499  5) 1500-1999  6) ≥2000 | RMB  If they cannot recall it, provide hints:  1) <200  2)200-499  3) 500-999  4) 1000-1499  5) 1500-1999  6) ≥2000 | RMB  If they cannot recall it, provide hints:  1) <200  2)200-499  3) 500-999  4) 1000-1499  5) 1500-1999  6) ≥2000 |
| 15 | Did you purchase any medication from a pharmacy? If so, how much did it cost? | RMB  If they cannot recall it, provide hints:  1) <30; 2) 30-49; 3) 50-99; 4)100-199; 5) 200-499;  6)500-999; 7) ≥1000. | | |
|  | Out-of-pocket | RMB  If they cannot recall it, provide hints:  1) <30; 2) 30-49; 3) 50-99; 4)100-199; 5) 200-499;  6)500-999; 7) ≥1000. | | |

| Section IV. Intangible Costs (Willingness to Pay) |
| --- |
| Besides direct medical and indirect costs, this illness may have caused physical suffering and psychological stress to you and your family. Assuming there were a way to completely eliminate such suffering and stress, what is the maximum amount you would be willing to pay? Amount: ______ RMB  Reference options:  ①<1000 ②3000元 ③5000元 ④8000元 ⑤10000元 ⑥15000元 ⑦20000元 ⑧Higher (specify) |

| **Thank you very much for your participation!** |
| --- |
